# An external ventricular drainage catheter impregnated with rifampicin, trimethoprim and triclosan, with extended activity against MDR Gram-negative bacteria: an *in vitro* and *in vivo* study

**DOI:** 10.1093/jac/dkz293

**Published:** 2019-07-13

**Authors:** Roger Bayston, Waheed Ashraf, Ivan Pelegrin, Katherine Fowkes, Alison S Bienemann, William G B Singleton, Ian S Scott

**Affiliations:** 1 School of Medicine, University of Nottingham, Nottingham, UK; 2 Infectious Diseases Department, Hospital Universitari de Bellvitge-IDIBELL, Barcelona, Spain; 3 Department of Neuropathology, Nottingham University Hospitals NHS Trust, Nottingham, UK; 4 Institute of Clinical Neurosciences, Faculty of Health Sciences, University of Bristol, Bristol, UK; 5 Department of Paediatric Neurosurgery, Bristol Royal Hospital for Children, Bristol, UK

## Abstract

**Background:**

External ventricular drainage (EVD) carries a high risk of ventriculitis, increasingly caused by MDR Gram-negative bacteria such as *Escherichia coli* and *Acinetobacter baumannii.* Existing antimicrobial EVD catheters are not effective against these, and we have developed a catheter with activity against MDR bacteria and demonstrated the safety of the new formulation for use in the brain.

**Objectives:**

Our aim was to determine the ability of a newly formulated impregnated EVD catheters to withstand challenge with MDR Gram-negative bacteria and to obtain information about its safety for use in the CNS.

**Methods:**

Catheters impregnated with three antimicrobials (rifampicin, trimethoprim and triclosan) were challenged in flow conditions at four weekly timepoints with high doses of MDR bacteria, including MRSA and *Acinetobacter*, and monitored for bacterial colonization. Catheter segments were also inserted intracerebrally into Wistar rats, which were monitored for clinical and behavioural change, and weight loss. Brains were removed after either 1 week or 4 weeks, and examined for evidence of inflammation and toxicity.

**Results:**

Control catheters colonized quickly after the first challenge, while no colonization occurred in the impregnated catheters even after the 4 week challenge. Animals receiving the antimicrobial segments behaved normally and gained weight as expected. Neurohistochemistry revealed only surgical trauma and no evidence of neurotoxicity.

**Conclusions:**

The antimicrobial catheter appears to withstand bacterial challenge for at least 4 weeks, suggesting that it might offer protection against infection with MDR Gram-negative bacteria in patients undergoing EVD. It also appears to be safe for use in the CNS.

## Introduction

External ventricular drainage (EVD) of CSF is widely used as a temporary measure in the management of raised intracranial pressure associated with a variety of conditions including trauma, haemorrhage, hydrocephalus, infection management and tumours. Patients requiring EVD are often in intensive care. Ventriculitis is a major complication of EVD. The reported incidence varies greatly, from 7.8% to 27%,^1–3^ but is usually 5%–10% of episodes. The variation in incidence can be due to differences in patient populations, EVD management regimens and particularly diagnostic criteria.

Many regimens have been proposed to reduce the rate of ventriculitis associated with EVD, ranging from antibiotic prophylaxis to ‘bundles’ of interventions. While the former shows some benefit, but a tendency to provoke resistance, bundles usually bring about a reduction in infection rates, but depend on sustained compliance, rates rising again after some time.^4^ Some changes have brought about clear benefit, such as tunnelling of the catheter and restricting CSF aspiration to that clinically necessary. However, following from the successful use of antimicrobial catheters in shunting, similar catheters have been introduced for EVD.^1^^,^^3^^,^^5–7^ Our catheters containing rifampicin and clindamycin (Bactiseal, Integra Life Sciences, Plainsboro, NJ, USA) have been successful in reducing staphylococcal infection,^8^ but they have no activity against Gram-negative bacteria. Though staphylococci are traditionally the main pathogens, recently there have been reports of increasing proportions of Gram-negative bacteria causing ventriculitis^9–11^ with an increase in multiresistance to antibiotics.^10–12^*Acinetobacter baumannii*, *Klebsiella pneumoniae* and *Enterobacter* spp. are the most commonly found Gram-negative bacteria. Using similar platform technology, we have developed an EVD catheter that retains activity against staphylococci while also having activity against most Gram-negative EVD pathogens, including MDR strains. The antimicrobials in the new catheter, rifampicin, trimethoprim and triclosan, were chosen partly for their antimicrobial spectrum and partly because of their chemical compatibility with the impregnation process. As well as reporting the *in vitro* activity of the catheter against these bacteria in rigorous clinically predictive tests, we have investigated the safety of the new catheter formulation for implantation into the brain using a rat model.

## Methods

### Processing of catheters

The method used was based on that already published.^13^ Briefly, medical grade silicone EVD catheter tubing (Vesta Inc., Franklin, WI, USA) ID 1.5 mm, OD 3.0 mm, was cut to 35 cm lengths. A solution of rifampicin (0.2%), trimethoprim (1%) and triclosan (1%) in chloroform was prepared and the catheter tubing was immersed in the solution at room temperature (∼22°C) for 1 h. The tubing was then removed and held in a current of air for ∼18 h for the solvent to evaporate. It was then briefly rinsed in ethanol to remove any surface drug accretions, dried and packaged, and sterilized by autoclaving at 121°C for 15 min. After sterilizing, three of the catheters were selected for quality assessment. They were immersed in chloroform for 1 h at room temperature to extract antimicrobials, then the extracts were analysed by HPLC-MS to confirm drug content.

### Test strains

All 17 bacteria tested were clinical isolates. They were: MRSA NB881, *Escherichia coli* NB2203 and NB2365, *Enterobacter cloacae* NB1454C and *K. pneumoniae* NB914 and F3990; a series of MDR strains: *A. baumannii* NB893, F1865, F2653 and F3859, *E. coli* F3986 (ESBL producing) and *E. coli* F3802 (NDM-1 producing); and a series of isolates from clinical cases where the rifampicin/clindamycin (Bactiseal) catheter had failed due to intrinsic resistance to either rifampicin or clindamycin or both: methicillin-resistant *Staphylococcus epidermidis* NB951, NB928, NB935 and F2364, and *Staphylococcus aureus* (MRSA) F1836. Their characteristics are shown in Table S1 (available as Supplementary data at *JAC* Online). The MICs of rifampicin and trimethoprim were determined using Etest (bioMérieux, Basingstoke, UK). The MIC of triclosan was determined by microtitre plate serial dilution.

### In vitro challenge

Antimicrobial and control EVD catheters were inserted aseptically into a modular challenge apparatus with capacity for 12 catheters to be tested simultaneously.^13^^,^^14^ The apparatus determines the ability of impregnated catheter tubing to withstand multiple bacterial challenges in flow conditions.

The catheters were maintained at 37°C while being perfused constantly with 20%, or 2% for *Acinetobacter*, tryptone soy broth (TSB; Oxoid Ltd, Basingstoke, UK) at a rate of 20 mL/h. At four timepoints (Days 0, 7, 14 and 21), the catheters were inoculated with 1 mL of 10^5^ cfu/mL suspension of the test bacteria. For each inoculation, flow was stopped and the catheters clamped, and, after injection of the inoculum into the test catheter, they remained clamped for 1 h to encourage bacterial attachment. A control catheter was set up for each test strain at each challenge, and changed when it became colonized. Each day, a sample of TSB was collected from each catheter, and viable cell counting was performed to determine bacterial numbers, by spreading 200 μL on a blood agar plate and incubating at 37°C for 48 h. All were tested in triplicate. Scanning electron microscopy was performed on segments of both control and processed catheters at the end of the challenge cycle. Segments of catheter 1.0 cm long were fixed in cold acetone overnight and then cut longitudinally and dehydrated with tetramethylsilane (Sigma–Aldrich), fixed to specimen stubs and sputter-coated with gold for 300 s before examination using a Jeol JSM 6060 microscope (Jeol Ltd, Tokyo, Japan).

### Neuroimplantation

Segments of the processed catheters were cut to 4 mm in length, packaged and sterilized as above. All animal work was performed in accordance with the UK Animal Scientific Procedures Act 1986 and was covered by both project and personal licences that were issued by the Home Office. Animal licences were reviewed and approved by the University of Bristol Ethics Committee (project licence PA95E951). All efforts were made to minimize animal use and suffering. Juvenile male Wistar rats (Harlan, UK) weighing 250 ± 5 g were group-housed in Techniplast 1500U cages with irradiated lignocel bedding and sawdust (International Product Supplies Ltd, UK). The study room was illuminated by fluorescent light set to give a cycle of 12 h of light and 12 h of dark, and was air-conditioned. The ambient temperature was held between 17°C and 22°C. Subjects were randomized into one of four groups (each containing four subjects) based on duration of treatment before sacrifice (7 and 28 days). Animals were individually anaesthetized with 2% inhaled isoflurane in oxygen in an anaesthetic chamber then placed in a stereotactic frame (David Kopf Instruments, Tujunga, CA, USA). Anaesthesia was maintained with inhaled 2% isoflurane/oxygen. The scalp fur was clipped and skin cleaned using alcoholic chlorhexidine. A midline incision from glabella to occiput exposing the skull was made, and a 5 mm burr hole was made 2 mm anterior and 2 mm lateral to the bregma on the right. The dura was opened and the catheter segment stereotactically placed in the frontal lobe on a stylet to a depth of 4 mm. The stylet was removed, leaving the segment in place. Bone wax was applied where needed for bone haemostasis, and the wound was closed with absorbable sutures (4/0 Vicryl Rapide^®^). Intramuscular analgesia was used post-operatively (buprenorphine, 30 μg/kg) and the animals were returned to their housing when recovered.

All animals were examined daily for clinical signs of toxicity or changes in behaviour, and body weight was recorded. All numerical data were analysed using GraphPad Prism^®^. According to the experimental protocol, any animals that displayed signs of neurological deterioration and/or weight loss greater that 10% of peak body mass would be terminated by Schedule 1 killing.

Groups 1 and 3 had control catheter segments implanted, and Groups 2 and 4 had impregnated catheter segments implanted. At the predetermined timepoints, animals were euthanized by anaesthetic overdose and then perfusion-fixed with 4% paraformaldehyde (Fisher Scientific, Loughborough, UK) in PBS (Oxoid), pH 7.4.

The brains containing the catheter segments were removed and sent for neuropathological examination. Catheters were removed from the brain post-mortem, after brain explantation and prior to neuropathological examination.

### Neuropathology

After noting the placement site, the catheter segments were removed from the brains, which were then processed and embedded in paraffin wax using standard laboratory protocols (PathCentre Tissue Processor: 3 day schedule). The neuropathologist carrying out the assessment (I. S. S.) was blind to group allocations. Sections were stained using haematoxylin and eosin (Leica ST5020 autostainer), and with antibodies raised against glial fibrillary acidic protein (GFAP) (Roche) (at a dilution of 1/5000), β-amyloid precursor protein (β-APP) (Roche) (1/20000) and neurofilament protein (NFP) (Roche) (1/50). Immunohistochemistry was performed on a Ventana Benchmark Ultra (Roche Diagnostics) automated stainer. The slides were dewaxed using cell conditioner 1 (Roche) at 95°C for 36 min and the primary antibodies were applied as follows: 24 min (GFAP), 32 min (NFP) and 36 min (β-APP). Haematoxylin counterstain was applied for 12 min. The slides were then mounted prior to viewing (Thermo Scientific ClearVue). Negative controls were performed by omitting the primary antibody. Positive controls were mounted on all slides having first demonstrated cross-reactivity between human and mouse tissues.

## Results

### Catheter drug content

Analysis of the impregnated catheters showed rifampicin 1.58 mg/g, trimethoprim 18.2 mg/g and triclosan 18.4 mg/g, all within the expected range.

### Test strain characteristics

Table S1 shows the 17 test strains and their susceptibilities to the three catheter drugs.

### In vitro challenge

**Figure 1.**
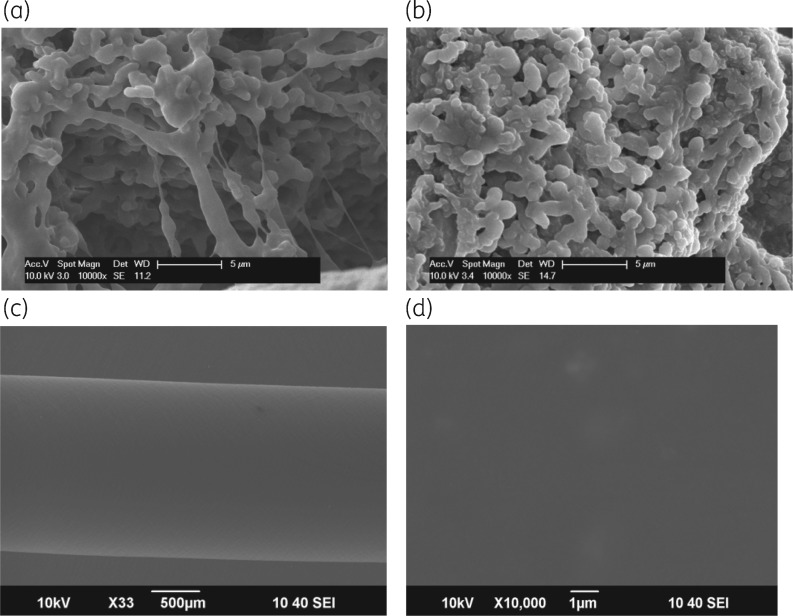
(a and b) Scanning electron micrographs (×10000) of the lumens of catheters after four weekly bacteria challenges followed by 6–7 days of perfusion. (a) Control catheter after challenge with *K. pneumoniae* NB914, at Day 7 of challenge 4. (b) Control catheter after challenge with MRSA F1836, at Day 7 of challenge 4. (c) Magnification of ×500. Lumen of the processed catheter after challenge with MRSA F1836, at Day 7 of challenge 4. The lower magnification was used to enable a more extensive field. (d) Magnification of ×10000. Lumen surface of (c) with no visible bacteria remaining. No bacteria were recovered after prolonged perfusion and agar culture.

In all cases, control catheters challenged with 10^5^ cfu/mL of the test bacteria colonized within a few days of inoculation, showing high bacterial counts (10^8^ cfu/mL). None of the impregnated catheters inoculated with the test bacteria became colonized after four successive weekly challenges. All control catheters colonized rapidly after inoculation and reached counts of ∼10^8^ cfu/mL within 6–7 days of inoculation, while counts in all the impregnated catheters declined to zero within 3 days, remaining at zero until the next challenge. From experiment, inoculation of 200 μL of effluent from the catheters onto blood agar plates incubated for 48 h was sufficient to determine absence of viable bacteria. This has been confirmed by scanning electron microscopic examination of impregnated catheters after 4 weeks of perfusion and bacterial challenge (Figure 1a–c). No re-growth occurred in the days after counts reached zero, nor was any resistance seen. Examples are shown in Tables 1 and 2 as all results were essentially similar.

**Table 1.** Results of four weekly challenges with MRSA F1836, showing on each challenge rising bacterial counts in the control catheter and no growth from the antimicrobial catheters

	Day 1	Day 2	Day 3	Day 6	Day 7
Day 0, challenge 1					
control	3.6 × 10^4^	3.9 × 10^5^	7.5 × 10^6^	4.8 × 10^7^	4.8 × 10^8^
test 1	0	0	0	0	0
test 2	0	0	0	0	0
test 3	0	0	0	0	0
Day 0, challenge 2					
control	2.8 × 10^4^	7.5 × 10^6^	1.5 × 10^7^	4.5 × 10^7^	1.1 × 10^8^
test 1	0	0	0	0	0
test 2	0	0	0	0	0
test 3	0	0	0	0	0
Day 0, challenge 3					
control	3.9 × 10^5^	3.6 × 10^6^	2.5 × 10^7^	2.1 × 10^7^	1.0 × 10^8^
test 1	0	0	0	0	0
test 2	0	0	0	0	0
test 3	0	0	0	0	0
Day 0, challenge 4					
control	2.5 × 10^5^	6.5 × 10^6^	6.6 × 10^7^	2.5 × 10^8^	1.1 × 10^8^
test 1	0	0	0	0	0
test 2	0	0	0	0	0
test 3	0	0	0	0	0

A value of 0 corresponds to a value that was below the lower limit of detection.

The negative cultures remained in the antimicrobial catheters throughout the four weekly challenges and each post-challenge perfusion (7 days).

**Table 2.** Results of four weekly challenges with *A. baumannii* NB893, showing on each challenge rising bacterial counts in the control catheter to the point where they became obstructed by biofilm by Day 7

	Day 1	Day 2	Day 3	Day 6	Day 7
Day 0, challenge 1					
control	1.3 × 10^7^	1.8 × 10^7^	2.9 × 10^8^	4.3 × 10^8^	blocked
test 1	0	0	0	0	0
test 2	0	0	0	0	0
test 3	0	0	0	0	0
Day 0, challenge 2					
control	2.8 × 10^7^	3.2 × 10^7^	2.3 × 10^7^	1.9 × 10^8^	blocked
test 1	80	5	0	0	0
test 2	275	65	0	0	0
test 3	65	15	0	0	0
Day 0, challenge 3					
control	2.7 × 10^7^	1.5 × 10^8^	5.4 × 10^8^	4.9 × 10^8^	blocked
test 1	225	0	0	0	0
test 2	155	0	0	0	0
test 3	185	0	0	0	0
Day 0, challenge 4					
control	2.7 × 10^7^	3.2 × 10^7^	5.0 × 10^7^	1.8 × 10^8^	blocked
test 1	160	0	0	0	0
test 2	115	0	0	0	0
test 3	105	0	0	0	0

A value of 0 corresponds to a value that was below the lower limit of detection.

There was no growth from the antimicrobial catheters at 24 h after the first challenge and though cultures were positive on monitoring on Day 1 after challenges 3 and 4, and Day 2 after challenge 2, they were negative by Day 3 and cultures remained negative thereafter in the antimicrobial catheters throughout the four weekly challenges and each post-challenge perfusion (7 days).

### Neuroimplantation

Both the 1 week and the 4 week groups of animals remained in good health, gaining weight as normal with no sign of neurotoxicity and showing normal grooming. All rats fed normally throughout. No difference was observed between the 1 week and the 4 week groups, or between controls and impregnated catheter groups. Weight gain for the 4 week cohort is shown in Figure S1.

### Neuropathology

**Figure 2.**
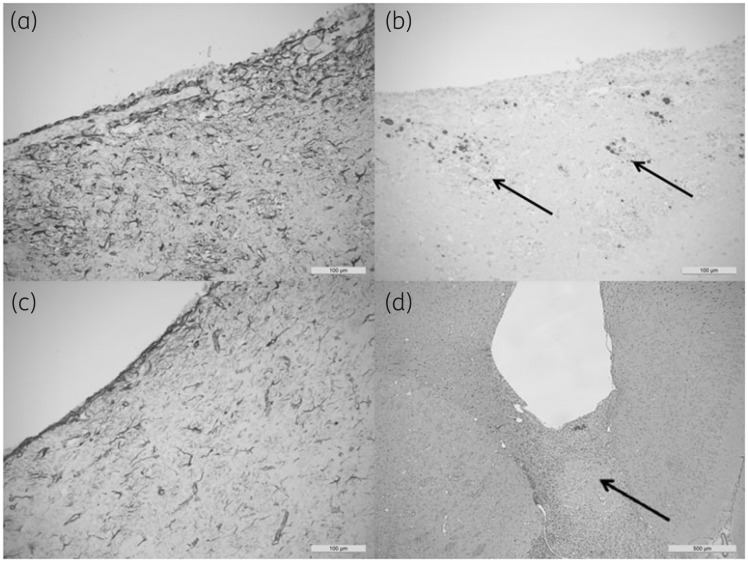
(a) Section showing the extent of reactive gliosis in the tract wall (GFAP) for a control catheter after 1 week. The surrounding tissue gliotic response, demonstrated in both the control and impregnated groups after 1 week, reflected surgical trauma at this site, which subsided after 4 weeks. (b) Stains for β-APP revealed traumatic axonal injury (deposits are indicated by arrows) within the walls of the tracts related to surgical trauma. This was present in all groups and varied in extent, but was independent of the contents of the catheter. (c) The gliotic response at 4 weeks (GFAP) was reduced in both the control and impregnated catheters. The traumatic axonal injury resulting from the catheter implant persisted at 4 weeks, but the extent of inflammation at 4 weeks was proportional to the degree of traumatic injury and there was no subjective evidence that the impregnated catheter induced either inflammation or gliosis in excess of that produced by the control catheter. (d) An example of a catheter (control) at 4 weeks (β-APP) showing an inflammatory infiltrate extending from the catheter tip to traverse the corpus callosum (arrow), demonstrating significant axonal pathology in the corpus callosum, which presumably resulted in traumatic vascular injury (not shown). These cases showed tract inflammation at 4 weeks regardless of the contents of the catheter segment tip.

Sections through the catheter tracts after 1 week and 4 weeks were compared histologically with both control catheters and impregnated catheters. The results are illustrated in Figure 2(a–d). After 1 week, the tracts in both control and impregnated groups showed tract inflammation associated with a fierce gliotic response (GFAP; Figure 2a). Stains for β-APP revealed minor axonal damage along the margins of the tract (Figure 2b). There was no subjective difference in the extent of inflammation between the control and impregnated groups at this stage unless there had been significant catheter trauma. After 4 weeks, the inflammatory infiltrate in the wall of the tract had subsided and the extent of the gliotic response was much reduced (GFAP; Figure 2c). The specimens all showed residual minor axonal injury within the tract, but the surgical inflammation appeared to have subsided. The inflammatory response was subjectively similar in both the control and impregnated groups after 4 weeks, and the surgical inflammation appeared to have resolved in comparison with the 1 week specimens. Examples were identified, from all experimental groups, in which there was significant traumatic axonal injury associated with catheter implantation (Figure 2d). These cases showed tract inflammation with diffuse cortical hypoxic injury illustrated by a ‘geographical’ pattern of cortical staining with β-APP and the presence of cortical ‘red’ neurons. These effects are, most probably, secondary to traumatic vascular injury. In these cases, the tract inflammation was directly proportional to the extent of surgical trauma and not the contents of the catheter.

## Discussion

Silicone EVD catheters impregnated with three antimicrobials were able to withstand successive weekly challenges with high numbers of MDR bacteria for at least 4 weeks. EVD catheters are not commonly used for more than 3 weeks. Activity against Gram-negative bacteria would be a significant advantage for an EVD catheter, extending its protective spectrum beyond its proven antistaphylococcal activity. Though failures due to intrinsically resistant strains of staphylococci in Bactiseal EVD catheters are rare,^15^ we have shown that the new formulation is active against those strains too. In addition, the results suggest that it will be equally effective against highly antibiotic-resistant strains that are now being increasingly encountered.

Most of the test bacteria were resistant to rifampicin, as expected. However, there is some evidence that, even when no conventional susceptibility is detected, rifampicin might have sublethal effects on the bacterial cell,^11^ and this has been confirmed clinically.^16^ We were also keen to employ the Dual Drug Principle,^17^^,^^18^ which indicates that this combination can be expected to reduce the likelihood of mutational resistance to any of the three drugs, and we previously have demonstrated this *in vitro*.^19^

The combination of antimicrobials, and particularly the triclosan component, has not been used in a neurosurgical setting before, though triclosan is used safely in other clinical applications such as biodegradable sutures^20^ and urinary catheter irrigation,^21^^,^^22^ and has been used safely in ureteral stents^23^ and central venous catheters.^24^ There is also a large amount of published human toxicology data on triclosan from transcutaneous, transmucous membrane and oral ingestion studies showing that it is safe.^25^ A pig femoral artery graft implantation study has also showed no inflammatory response over and above that of control catheters.^26^ We have previously demonstrated its lack of toxicity in the rat peritoneal cavity.^19^ We therefore implanted catheter segments into the brains of a series of rats. Rats in the first series were euthanized at 1 week to determine the effect of surgical trauma, and rats in the second series were euthanized at 4 weeks to determine any inflammation or neurotoxicity that might be caused by the antimicrobials. Importantly, both series of rats receiving the antimicrobial catheter segments gained weight normally and displayed no signs of illness throughout, and were indistinguishable in these respects from the control animals. When explanted brains were examined neuropathologically, though the relatively large segments caused considerable surgical trauma, there was no evidence of inflammation or toxicity that could be attributed to the antimicrobials in the catheter. We were pleased to note that US and EU regulators have recently banned triclosan in all but medical products, recognizing its importance and safety in this area.

### Conclusions

The antimicrobial EVD catheter therefore appears to be effective against EVD pathogens and safe for implantation into the brain. The relatively long duration of protection against colonization by even MDR bacteria suggests that it might be beneficial in reducing EVD infections. Further studies will include insertion of whole catheters into a large animal model and bacterial challenge *in vivo*. If this study is successful, human studies will follow.

## Funding

This work was supported by an MRC Confidence in Concept grant (CiC2017004).

## Transparency declarations

R. B. is holder of patents on the process described, assigned to his university, with no personal gain. All other authors: none to declare.

## Supplementary Material

dkz293_Supplementary_Data

## References

[1] Babu MA, Patel R, Marsh WR et al Strategies to decrease the risk of ventricular catheter infections: a review of the evidence. Neurocrit Care 2011; 16: 194–202. 10.1007/s12028-011-9647-z 22045248

[2] Wong GKC, Poon WS, Wai S et al Failure of regular external ventricular drain exchange to reduce cerebrospinal fluid infection: result of a randomised controlled trial. J Neurol Neurosurg Psychiatr 2002; 73: 759–61. 10.1136/jnnp.73.6.759 PMC1757349 12438486

[3] Wright K, Young P, Brickman C et al Rates and determinants of ventriculostomy-related infections during a hospital transition to use of antibiotic-coated external ventricular drains. Neurosurg Focus 2013; 34: E12. 23634916 10.3171/2013.2.FOCUS12271

[4] Dasic D, Hanna SJ, Bojanic S et al External ventricular drain infection: the effect of a strict protocol on infection rates and a review of the literature. Br J Neurosurg 2006; 20: 296–300. 17129877 10.1080/02688690600999901

[5] Zabramski JM, Whiting D, Darouiche RO et al Efficacy of antimicrobial-impregnated external ventricular drain catheters: a prospective, randomized, controlled trial. J Neurosurg 2003; 98: 725–30. 12691395 10.3171/jns.2003.98.4.0725

[6] Gutierrez-Gonzalez R, Boto GR. Do antibiotic-impregnated catheters prevent infection in CSF diversion procedures? Review of the literature. J Infect 2010; 61: 9–20. 20363252 10.1016/j.jinf.2010.03.030

[7] HarropJS, SharanAD, RatliffJ et al Impact of a standardized protocol and antibiotic-impregnated catheters on ventriculostomy infection rates in cerebrovascular patients. Neurosurgery2010; 67: 187–91.2055910510.1227/01.NEU.0000370247.11479.B6

[8] Thomas R, Lee S, Patole S et al Antibiotic-impregnated catheters for the prevention of CSF shunt infections: a systematic review and meta-analysis. Br J Neurosurg 2012; 26: 175–84. 21973061 10.3109/02688697.2011.603856

[9] Hogg GM, Barr JG, Webb CH. In-vitro activity of the combination of colistin and rifampicin against multidrug-resistant strains of *Acinetobacter baumannii*. J Antimicrob Chemother 1998; 41: 494–5. 9598783 10.1093/jac/41.4.494

[10] Kim B-N, Peleg AY, Lodise TP et al Management of meningitis due to antibiotic-resistant *Acinetobacter* species. Lancet Infect Dis 2009; 9: 245–55. 19324297 10.1016/S1473-3099(09)70055-6 PMC2760093

[11] Lee HJ, Bergen PJ, Bulitta JB et al Synergistic activity of colistin and rifampin combination against multidrug-resistant *Acinetobacter baumannii* in an *in vitro* pharmacokinetic/pharmacodynamic model. Antimicrob Agents Chemother 2013; 57: 3738–45. 23716052 10.1128/AAC.00703-13 PMC3719722

[12] Hsueh PR, Chen WH, Luh KT. Relationships between antimicrobial use and antimicrobial resistance in Gram-negative bacteria causing nosocomial infections from 1991–2003 at a university hospital in Taiwan. Int J Antimicrob Agents 2005; 6: 463–72. 10.1016/j.ijantimicag.2005.08.016 PMC7126312 16280243

[13] Bayston R, Grove N, Siegel J et al Prevention of hydrocephalus shunt catheter colonisation in vitro by impregnation with antimicrobials. J Neurol Neurosurg Psychiatr 1989; 52: 605–9. 10.1136/jnnp.52.5.605 PMC1032173 2732730

[14] Bayston R, Lambert E. Duration of protective activity of cerebrospinal fluid shunt catheters impregnated with antimicrobial agents to prevent bacterial catheter-related infection. J Neurosurg 1997; 87: 247–51. 9254088 10.3171/jns.1997.87.2.0247

[15] Bayston R, Ashraf W. Antibiotic resistant infections with antibiotic-impregnated Bactiseal catheters for ventriculoperitoneal shunts. Br J Neurosurg 2011; 25: 780. 22115016 10.3109/02688697.2011.633641

[16] Bassetti M, Repetto E, Righi E et al Colistin and rifampicin in the treatment of multidrug-resistant *Acinetobacter baumannii* infections. J Antimicrob Chemother 2008; 61: 417–20. 18174197 10.1093/jac/dkm509

[17] Ehrlich P. Pathology in therapeutics: scientific principles, methods and results. Lancet 1913; 445–51.

[18] Zhao X, Drlica K. Restricting the selection of antibiotic-resistant mutants: a general strategy derived from fluoroquinolone studies. Clin Infect Dis 2001; 33 Suppl 3: S147–56. 11524712 10.1086/321841

[19] Bayston R, Fisher LE, Weber K. An antimicrobial modified silicone peritoneal catheter with activity against both Gram-positive and Gram-negative bacteria. Biomaterials 2009; 30: 3167–73. 19289248 10.1016/j.biomaterials.2009.02.028

[20] Barbolt TA. Chemistry and safety of triclosan, and its use as an antimicrobial coating on coated Vicryl Plus antibacterial suture (coated polyglactin 910 suture with triclosan). Surg Infect (Larchmt) 2002; 3 Suppl 1: S45–53. 12573039 10.1089/sur.2002.3.s1-45

[21] Holroyd S. A new solution for indwelling catheter encrustation and blockage. J Community Nurs 2017; 31: 48–52.

[22] Pannek J, Vestweber AM. Clinical utility of an antimicrobial blocking solution in patients with an indwelling catheter. Aktuelle Urol 2011; 42: 51–4. 21267806 10.1055/s-0030-1262758

[23] Cadieux PA, Chew BH, Nott L et al Use of triclosan-eluting ureteral stents in patients with long-term stents. J Endourol 2009; 23: 1187–94. 19538062 10.1089/end.2008.0437

[24] Silva Paes Leme AF, Ferreira AS, Alves FA et al An effective and biocompatible antibiofilm coating for central venous catheter. Can J Microbiol 2015; 61: 357–65. 25826042 10.1139/cjm-2014-0783

[25] Jones RG, Jampani HB, Newman JL et al Triclosan: a review of effectiveness and safety in health care settings. Am J Infect Control 2000; 28: 184–96. 10760227

[26] Hernandez-Richter T, Schardey HM, Löhlein F et al The prevention and treatment of vascular graft infection with a Triclosan (Irgasan)-bonded Dacron graft: an experimental study in the pig. Eur J Vasc Endovasc Surg 2000; 20: 413–8. 11112458 10.1053/ejvs.2000.1199

