# Supplementary material for: An external ventricular drainage catheter impregnated with rifampicin, trimethoprim and triclosan, with extended activity against MDR Gram-negative bacteria: an in vitro and in vivo study
Source: J Antimicrob Chemother. 2019 Jul 13;74(10):2959–64. doi: 10.1093/jac/dkz293 (PMC6753475; doi:10.1093/jac/dkz293)
Supplement: dkz293_Supplementary_Data [file dkz293_supplementary_data.docx]

**Supplementary data**

**Table S1.** The 17 test strains and their susceptibilities (MIC) to the three catheter drugs

| **Isolates** | **Rifampicin (mg/L)** | **Trimethoprim (mg/L)** | **Triclosan (mg/L)** |
| --- | --- | --- | --- |
| MRSA - NB881 | 0.012 | 0.25 | 0.004 |
| *E coli* - NB2203 | >32 | 0.25 | 0.008 |
| *E coli* - NB2365 | >32 | 0.25 | 0.008 |
| *Enterobacter cloacae* - NB1454C | >32 | 0.25 | 0.08 |
| *Klebsiella pneumoniae* - NB914 | >32 | 0.25 | 0.2 |
| *Klebsiella pneumoniae* - F3990 | >32 | 0.25 | 0.2 |
| *Acinetobacter baumannii* - NB893 | >32 | >32 | 0..4 |
| *Acinetobacter baumannii* - F1865 | >32 | >32 | 1 |
| *Acinetobacter baumannii* - F2653 | >32 | >32 | 1 |
| *Acinetobacter baumannii* - F3859 | >32 | >32 | 2 |
| *E coli* - F3986 (ESBL) | >32 | 0.25 | 0.008 |
| *E coli* - F3802 (NDM-1) | >32 | 0.25 | 0.008 |
| MRSE - NB951 | >32 | 0.125 | 0.004 |
| MRSE - NB928 | >32 | 0.125 | 0.004 |
| MRSE - NB935 | >32 | 0.125 | 0.004 |
| MRSE - F2364 | >32 | 0.25 | 0.004 |
| MRSA - F1836 | >32 | 0.25 | 0.004 |

NB and F are the laboratory registry designations





**Figure S1.** Weight gain during 4 weeks of catheter segment implantation, in animals with either plain or impregnated catheters. All animals gained weight at the rate expected in non-operated subjects and there was not difference between the two groups.
